# The Effects of Multi-Sociodemographic Characteristics of Construction Sites Personnel on Perceptions of Safety Climate-Influencing Factors: The Construction Industry in Saudi Arabia

**DOI:** 10.3390/ijerph18041674

**Published:** 2021-02-09

**Authors:** Ibrahim Mosly, Anas A. Makki

**Affiliations:** 1 Department of Civil Engineering, Faculty of Engineering—Rabigh Branch, King Abdulaziz University, Jeddah 21589, Saudi Arabia; 2 Department of Industrial Engineering, Faculty of Engineering—Rabigh Branch, King Abdulaziz University, Jeddah 21589, Saudi Arabia; nhmakki@kau.edu.sa

**Keywords:** safety climate, construction, perceptions, Saudi Arabia, sociodemographic characteristics, factors

## Abstract

The construction industry in Saudi Arabia relies prominently on migrant workers of multi-sociodemographic characteristics with different perceptions of a safety climate. The exploration of the perceptions regarding the safety climate among various groups of migrant workers may help identify effective means of improving safety levels at construction sites in Saudi Arabia. This study aimed to examine the effects of multi-sociodemographic characteristics of construction site personnel on their perceptions of the factors that influence the safety climate at construction sites in Saudi Arabia. Data were collected from 401 construction site workers, employed at ongoing construction project sites in Saudi Arabia, using a designed questionnaire. A generalized, linear model approach was applied, using the single ordinal logistic regression method, to analyze the collected data. The results revealed the significant sets of sociodemographic characteristics and their associated subgroups that had significant effects on the perception of importance assigned to each safety climate-influencing factor. These findings provide a better understanding of the views of construction site personnel on the safety climate and can assist construction industry decision-makers, safety policy designers, government agencies, and stakeholders when designing better-targeted enhancement plans and strategies to improve the safety climate of construction sites, based on the sociodemographic makeup of the personnel at each construction site.

## 1. Introduction

Construction is considered to be a high-risk industry that is subjected to work-related illnesses, injuries, and deaths [1], and is associated with some of the highest reported occurrences of occupational accidents [2]. Construction often includes the performance of highly hazardous tasks [3]. Construction personnel who are subjected to these risks and hazards require particular safety conditions. Many work-related illnesses and injuries can be prevented if proper preventative strategies are in place, and the key factors resulting in these hazardous events must be studied to determine how they can be prevented. This task represents one of the most significant objectives of the construction industry—to enhance occupational health and safety [4]. Construction workers are the most vulnerable members of the construction industry, exposed to greater occupational health and safety hazards than other construction personnel. Safety management represents a significant emerging topic in the construction industry [5] and is necessary to protect construction personnel. Further exploration of this area remains constantly necessary to enhance the safety of construction industry personnel.

Although digital technology enhancements can play a role in improving construction safety management, issues associated with human factors remain relevant, including individual safety perceptions, attitudes, and behaviors [6]. Thus, a large factor during any safety improvement strategy relies on human-to-human development and the changing of safety perceptions. The decisions made by employees regarding hazards and accidents are affected by related variables such as the frequency and severity, and identification ability [7]. Therefore, worker experience plays a large role in determining safety perceptions. People take actions based on their perceptions; thus, the perception of safety affects employees′ behavior [8], revealing an important association between perception and behavior. The negative influence of high-risk tolerance on safety behavior can be neutralized by introducing a positive safety climate [9]. Furthermore, a strong positive relationship was identified between injury levels and safety climate levels [10]. Construction companies with enhanced safety climates are likely to employ construction workers with high-risk perception [11]. Thus, increasing safety climate levels appears to represent an important step toward enhancing overall safety performance [12]. The safety climate affects both hazard recognition and safety risk perception [13]. Owners, contractors, and specialty contractors can benefit from an improved safety climate, which can result in attitudes and perceptions that contribute to improved safety performance [14]. Moreover, the aspect of safety climate perception has been positively associated with increased job satisfaction levels [15,16]. Construction employees who discuss safety issues with their supervisors in a more contented manner are more likely to sense that safety is appreciated in their workplace, are more experienced conducting tasks safely, and willingly assist in the creation of a safe workplace [17]. Safety perception may be positively affected by increased supervisor contact [18]. In addition, safety perception can also be influenced by co-workers. Compared with the safety climate imposed by supervisors, the safety climate imposed by co-workers can strongly influence safety behavior, specifically safety participation, at both the individual and group levels [19]. Furthermore, the safety climate imposed by co-workers encourages the establishment of safety behaviors and can help workers develop a positive workplace risk perception [20]. To effectively influence the safety climate perception, successful communication is necessary, whether with a supervisor or a co-worker. Better safety climate perceptions can be achieved by clearly communicating the rules [9]. Unanticipated injuries can occur when inadequate communication occurs among workers regarding safety hazards and suitable injury avoidance measures [21]. Supervisors must communicate high safety goals; otherwise, the influence of senior managers may appear unimportant [22].

Due to labor shortages in the construction industry, many countries employ ethnic minority (EM) or migrant workers, who are more likely to be subjected to fatal and nonfatal occupational injuries compared with local workers [23]. Employing workers from different nationalities, who speak different languages, can result in the development of communication issues and wide differences in safety climate perceptions. In several developed countries, the fatality rate among EM workers is higher than that of local workers, according to unofficial statistics [24], which reflects the imbalance among safety climate perception between local and EM workers at construction sites. To improve the safety of migrant workers, the safety climate should be significantly considered [25]. Due to the difficulties in contacting EM workers and measuring their perceptions, they are often underrepresented in safety climate research [26]. Thus, more in-depth research regarding the safety climate perceptions of EM construction workers should be conducted, as they represent the majority of construction workers, in many countries. Research examining the ethnic differences in safety and health results has indicated that these differences could explain differences in work-related factors [27]. In the United States of America, the safety climate level among Hispanic construction workers is lower than that among non-Hispanics construction workers, indicating the reduced importance of safety among the Hispanic workers [28].

Safety climate boundaries are not uniform across organizations and must be evaluated differently for various groups of employees [29]. The safety climate perceptions of construction workers can be shaped at various levels and differ between workgroups [30]. Several factors can affect these differences in safety climate perceptions among groups. Factors, such as nationality, marital status, the number of family members, and drinking habits, can considerably alter safety climate perceptions [26]. Furthermore, individual risk perceptions can be forecasted by organization-, group-, and individual-level factors [31]. Disparities in trade safety climate perceptions exist and may be associated with changes in tasks, environment, and exposure to risk [32]. For instance, the safety climate perception of management-level staff is higher than that of labor-level staff [33]. Similarly, different trades within the construction industry may not share the same risks and hazards. For instance, painters are exposed to different types of hazards than electricians. The expansion and implementation of effective methods designed to alleviate workplace hazards could be difficult due to differences in the safety perceptions of various hierarchical groups within an institution [34]. A safety professional’s ability to create and implement safety programs improves when the underlying reasons for these differences in safety climate perceptions among subgroups are better understood [35].

The Saudi Arabian construction industry relies heavily on migrant workers from various nationalities. Due to the low safety performance and the presence of many hazards at construction sites in Saudi Arabia [36], investigating safety climate perceptions among construction workers in Saudi Arabia is essential for improving safety outcomes. Three studies were previously conducted to explore different aspects of safety climate perceptions among construction workers in Saudi Arabia. The first study determined the current situation and identified a set of 13 factors influencing the safety climate in the construction industry of Saudi Arabia [37]. The second study revealed that the 13 factors act as determinants of safety climate in construction sites of Saudi Arabia under three key components of safety climate which are safety commitment, safety interaction, and safety support [38]. The third study developed a safety climate prediction model and revealed the set of significant safety climate predictors in construction sites of Saudi Arabia [39]. To further enrich the area of research related to safety climate workers′ perceptions in the Saudi Arabian construction industry, this study aimed to determine the perceptions and multi-sociodemographic characteristics of construction site personnel regarding the factors that influence the safety climate. The results of this study can be used as a reference point for examining the safety climate perception of different workers in Saudi Arabia and provide significant information for different construction industry stakeholders for use when designing strategies and plans, as well as during policy and decision making.

## 2. Materials and Methods

The primary objective of this study was to reveal the significant perceptions and multi-sociodemographic characteristics of construction site personnel regarding the various factors that influence the safety climate of the construction industry in Saudi Arabia. Therefore, a survey design was used to obtain a wide range of opinions in an attempt to capture their perceptions for the quantitative measurement that formed the used dataset. The dataset includes information collected from 401 construction site personnel, using a designed questionnaire survey, site visits, face-to-face interviews, and Google Forms for data-entry, dataset storage and initial setup. The questionnaire included questions asking construction site personnel to select the group for each of the sociodemographic characteristics that best represented them, as summarized in Table 1. A total of six sociodemographic characteristics were chosen in this study, which were age, nationality, education, occupation, years of experience, and trade specialty. The age characteristic was expressed in non-overlapping year ranges, nationality was expressed in citizenship groups, education was expressed in the highest level of qualification attained, the occupation was expressed in the general job domains of participants, years of experience was expressed in ranges of non-overlapping years spent in the construction profession, and trade specialty was expressed in specific construction activities. These sociodemographic characteristics were used based on several relevant studies [5,20,24,28,32,33,34] and were found to be the most applicable to the Saudi Arabian construction environment. Other sociodemographic characteristics which have been used in many studies, such as alcohol consumption, were not considered due to being inapplicable to the Saudi Arabian construction industry, as drinking is considered prohibited in the country. Construction site personnel were also asked to rate their perceptions regarding the importance of each of the 13 safety climate-influencing factors, which are listed in Table 2. Their perceptions were collected on a 5-point Likert scale (i.e., 5 = extremely important, 4 = important, 3 = neither, 2 = unimportant, 1 = extremely unimportant). The cross-sectional data were collected during the period 2019–2020, from the active construction sites of large projects in Saudi Arabia. To ensure the representativeness of the construction industry of Saudi Arabia in the collected information, those construction sites from which the data were collected were selected based on a set of criteria. The criteria are the availability of those sites during the time of data collection, being active sites, their project size being large, and the diversity of educational backgrounds, professional and trade specialty composition, and the multinationalism of their construction site personnel. Through a sampling frame, construction site personnel working at the selected project sites were targeted with an equal chance to take part in the questionnaire and a random sample of 401 employees responded and fully complete the questionnaire.

Using a quantitative research design and a random sampling data collection process, the objective of this study was attained through the application of an inferential statistical design. Each of the 13 safety climate-influencing factors was examined as dependent variables (DVs) against each of the sociodemographic characteristics of construction site personnel, which represented the independent variables (IVs). The importance of each of the 13 factors was rated by construction site personnel, using the above-described 5-point Likert scale; therefore, these 13 factors were treated as ordinal, categorical DVs. The sociodemographic characteristics were treated as either ordinal categorical (i.e., age, education, years of experience) or nominal categorical (i.e., nationality, occupation, and trade specialty) IVs. Therefore, for such a statistical design, a generalized linear model approach was applied, using the single ordinal logistic regression method.

The ordinal, logistic regression is a maximum-likelihood, estimation-based method, represented as a proportional odds model that uses a logit link function to executes logarithmic transformations of cumulative probabilities to express non-linear relationships between the DV and IVs into a linear model [40,41,42]. Therefore, each of the safety climate-influencing factors was regressed against each of the sociodemographic characteristics (i.e., a series of 13 × 6 = 78 models) using the Statistical Package for Social Sciences computer software (SPSS version 23.0) [43]. This regression revealed the set of sociodemographic characteristics associated with the perception ratings that significantly influenced the relative importance of each safety climate-influencing factor. The Omnibus test (likelihood ratio *χ*^2^) was conducted to assess the significance level (*p* < 0.05, *p* < 0.01, *p* < 0.001) of the contribution of each sociodemographic characteristic to each safety climate-influencing factor. Furthermore, the pseudo-Nagelkerke′s *R*^2^ value was used to measure the strength of the association between each safety climate-influencing factor and each sociodemographic characteristic. To verify the ordinal logistic regression assumptions of proportionality or the parallelism of the odds, a test of parallel lines (–2 log-likelihood *χ*^2^) was conducted. An insignificant (i.e., *p* > 0.05) result indicates that the location parameters of the regression model coefficients are the same across all DV categories. The Wald *χ*^2^ statistic was used to assess the significance of each estimated regression model coefficient (*β*) associated with each category (i.e., location) and pertaining to a sociodemographic characteristic for the prediction of the importance of a given safety climate factor. This significance was measured at a 95% Wald confidence interval for their associated odds ratio [Exp(*β*) or *e^β^*]. Odds ratios were used to interpret the results in terms of quantifying the effect of each sociodemographic group on the perception of each safety climate-influencing factors. An odds ratio with a value greater than 1 [Exp(*β*) > 1] indicates that a sociodemographic group is most likely to rate an examined safety climate-influencing factor as more important than the reference group used for that particular sociodemographic characteristic. Inversely, an odds ratio of less than 1 [Exp(*β*) < 1] indicates that a sociodemographic group is likely to rate a given safety climate-influencing factor as being less important than the reference group. Reference groups are the sociodemographic characteristic groups to which other groups will be compared. Table 3 shows the assignment criteria used to designate the reference groups for each of the 6 sociodemographic characteristics in this study. By default, the ordinal, categorical, sociodemographic characteristics (age, education, and years of experience) or the highest order category (last category on an ascending scale) were assigned as the reference groups. However, the reference groups for the nominal, categorical, sociodemographic characteristics (nationality, occupation, and trade specialty) were assigned theoretically, based on the sample in this study, and the added value of the interpretation of results aimed to achieve the best possible representation of the construction industry in Saudi Arabia. Thus, the Pakistani nationality was designated as the reference group in the sample. The construction site personnel occupation of a manager was assigned as the reference group for the occupation sociodemographic characteristic because this position generally has the highest level of authority and the highest educational level among construction site personnel. Similarly, the construction site personnel classified under the trade specialty of safety and quality control were assigned as the reference group for the trade specialty sociodemographic characteristic because they usually have the highest educational level among construction site personnel and their daily work is directly related to safety; therefore, observing how other trade specialty groups compare against this group is of particular interest.

Although generated classification models are relatively capable of predicting the probability of the importance ratings for each safety climate-influencing factor, by using the significant sociodemographic characteristics and its associated groups as predictors, forecasting and rating classification was not the objective of this study. This study was concerned with the estimated location parameters of regression model coefficients and their associated odds ratio relative to a reference group. Therefore, more focus was given to the interpretation of estimated location parameters, instead of the threshold estimates or the intercepts of the regression models. The results and discussions of the analysis are presented subsequently.

## 3. Results and Discussion

In this section, significant effects among different construction personnel regarding safety climate factors are calculated and discussed, which will facilitate the recognition of different levels of safety climate perception among various construction site personnel and assist construction industry stakeholders in the development of methods for enhancing safety levels at construction sites. A series of 78 ordinal logistic regression models were generated, as described above. This allowed each sociodemographic characteristic to be examined against each of the safety climate-influencing factors. A total of 33 out of 78 models demonstrated significance and were analyzed further. Only statistically significant models were considered for interpretation. The resulting significant models can be found in the accompanying Supplementary Materials. These 33 models, representing significant sociodemographic characteristics and their associated groups, are presented in 13 tables (Tables S1–S13). Each table refers to one safety climate-influencing factor, ranked as shown in Table 2. The resulting significant sociodemographic characteristics associated with each studied factor were ranked based on their relative ability to explain variation in the importance ratings of the studied factor, using Nagelkerke′s *R*^2^. Table 4 provides a summary of the findings in Tables S1–S13. In the next subsections, results and discussions on the effects of the significant sociodemographic characteristics of construction site personnel on the perceptions of each of the safety climate-influencing factors are presented.

### 3.1. Supervision, Guidance, and Inspection

The results presented in Table 4 and Table S1 revealed that trade specialty, education, and nationality were the significant sociodemographic characteristics that influenced the perceived importance of supervision, guidance, and inspection as a safety climate-influencing factor. The results showed that carpenters and plumbers were significantly more likely to rate supervision, guidance, and inspection as being less important compared with the reference group of personnel working in the field of safety and quality control. The low level of importance attributed to supervision, guidance, and inspection by carpenters and plumbers may be due to their perception that their specialties involve simple, repetitive tasks that do not require high levels of supervision, guidance, and inspection. Additionally, these trades may attribute less importance to supervision, guidance, and inspection because this level of worker tends to be less educated than individuals among the safety and quality control staff. Moreover, the results showed that construction personnel with educational levels ranging from illiterate to diploma holder were more likely to rank supervision, guidance, and inspection as being less important than personnel with bachelor′s degrees, which may reflect the limited awareness and background knowledge of less-educated groups regarding existing construction hazards and the importance of supervision, guidance, and inspection for mitigating these dangers. Furthermore, Egyptian personnel was the most likely nationality to rate supervision, guidance, and inspection as being important relative to the reference group of Pakistani nationality. In contrast, personnel belonging to the Bangladeshi nationality were the most likely to rate supervision, guidance, and inspection as being less important than those of Pakistani nationality. This result indicates that Egyptian construction personnel recognizes the importance of supervision, guidance, and inspection on the determination of the safety climate, whereas Bangladeshi construction personnel requires more attention and training in this matter. This finding might be attributed to cultural background differences between these nationality groups (e.g., individualistic versus collectivist, and low- versus high-power-distance cultures) and how they perceive the importance of supervision, guidance, and inspection in the safety climate context.

### 3.2. Appraisal of Risks and Hazards

For the appraisal of risks and hazards as a safety climate-influencing factor, the results presented in Table 4 and Table S2 show that education and age are the significant sociodemographic characteristics that determine the importance of this factor. The results demonstrating personnel with educational levels ranging from illiterate to secondary were significantly more likely to rate the appraisal of risks and hazards as being less important than personnel with bachelor′s degrees. This result may reflect the limited awareness and background knowledge of less-educated groups regarding construction hazards and risks and the importance of appraisal for mitigating these risks. Furthermore, the results showed that the youngest personnel group, aged between 18 and 20 years, was the only group that was significantly likely to rate this factor as being less important than the eldest personnel group, aged older than 50 years. These individuals may be less educated and less experienced due to their younger ages.

### 3.3. Social Security and Health Insurance

The results presented in Table 4 and Table S3 revealed that education was the only significant sociodemographic characteristic to influence the importance of social security and health insurance as a safety climate-influencing factor. The illiterate personnel group was significantly more likely to rate social security and health insurance as less important than bachelor′s degree holders, which may be due to their limited background knowledge and a lack of awareness regarding their job rights.

### 3.4. Workmate Influence

The results presented in Table 4 and Table S4 showed that nationality and education were the significant sociodemographic characteristics associated with the importance of the workmate influence factor. Personnel of Bangladeshi and Yemeni nationalities were more likely to rate the workmate influences as being less important than the personnel of Pakistani nationality. In contrast, employees of Egyptian and Filipino nationalities were more likely to rate workmate influences as being more important than personnel of Pakistani nationality. This result is likely due to cultural background differences between those nationality groups (e.g., individualistic versus collectivist, and low- versus high-power-distance cultures) and how they perceive the importance of workmate influence in a safety climate context. Moreover, the results demonstrated the significance of educational levels, with personnel ranging from illiterate to those with secondary education, who were most likely to rate workmate influences as being less important than bachelor′s degree holders. This result might be due to their limited background knowledge and awareness of the effects of workmate′s influences on behavior and the safety climate in a working environment. Another study examined the significance of considering co-workers’ actual safety responses, which is an element of workmate influence and represents a dimension of a group-level safety climate factor in the construction industry [44]. To enhance the safety climate, workers should be positively encouraged to advise their colleagues on safety-related issues, which can result in the development of a pleasant, trusting, and safer work environment [45].

### 3.5. Management Safety Justice

The results presented in Table 4 and Table S5 revealed that trade specialty, education, and experience are the significant sociodemographic characteristics that influence the importance of management safety justice as a safety climate-influencing factor. The results showed that administration personnel and plumbers are the most likely trade specialty groups to rate management safety justice as being more important than construction site personnel working in the field of safety and quality control. The administration staff are usually office-based staff who are usually perceived as being less exposed to hazards and risks than other construction site personnel. However, the observation that plumbers rate management safety justice as being more important than safety and quality control staff is an interesting result. Trade specialties often do not perceive themselves as being fairly and justly empowered by management to influence the aspects relevant to their safety or to manage health and safety incidents, risks, and hazards. Therefore, both groups express a lack of empowerment by management. Moreover, the results showed that personnel with educational levels ranging from illiterates to diploma holders were more likely to rate management safety justice as being less important than bachelor′s degree holders. This finding may be due to their limited background knowledge and the lack of awareness regarding the importance of management safety justice. Additionally, these groups may not be expecting any level of empowerment to influence aspects relevant to their safety or the ability to manage health and safety incidents, risks, and hazards. Furthermore, midcareer personnel with 11–15 years of experience were more likely to rate management safety justice as being more important than the end-of-career personnel group with 20 years or more of experience. Midcareer personnel are both highly experienced and have considerably long careers in their futures. Therefore, they may perceive themselves as being trustworthy and deserving of empowerment by management to influence aspects that are relevant to their safety and to manage health and safety incidents, risks, and hazards on construction sites.

### 3.6. Management Commitment to Safety

The results presented in Table 4 and Table S6 show that education and nationality are the significant sociodemographic characteristics that influenced the importance of management commitment to safety. The results showed that personnel with educational levels ranging from illiterate to diploma holder were more likely to rate the management commitment to safety as being less important than bachelor′s degree holders, which may be due to limited awareness and background knowledge and the failure to recognize the vital role played by management commitment to safety and safety enforcement. These groups may perceive management as not working directly at construction sites and, therefore, their involvement in safety is not considered to be important to the safety climate. Moreover, a personnel group of a Bangladeshi nationality was more likely to rate the management commitment to safety as being less important than the personnel of Pakistani nationality. In contrast, the personnel group of Egyptian nationality was more likely to rate this factor as being more important than the personnel of Pakistani nationality. Differences in perceptions among personnel of Egyptian, and Bangladeshi, and Pakistani nationalities are consistent with the results observed for other factors. Therefore, these differences are likely due to cultural background differences among these nationality groups (e.g., individualistic versus collectivist, and low- versus high-power-distance cultures).

### 3.7. Education and Training

The results presented in Table 4 and Table S7 revealed that nationality, education, and occupation were the significant sociodemographic characteristics that influenced the importance of education and training as an influential safety climate factor. Personnel of Bangladeshi and Somali nationalities were likely to rate education and training as being less important than the personnel of Pakistani nationality. In contrast, the personnel group of Egyptian nationality was more likely to rate this factor as more important than personnel of Pakistani nationality. Unfortunately, education and training are well documented in the literature as having important impacts on construction workers′ safety. The lack of recognition of the importance of these factors among personnel of Bangladeshi and Somali nationalities is a matter of concern. Moreover, personnel with educational levels ranging from illiterate to diploma holders attributed less importance to education and training, which is likely due to their limited background knowledge. Furthermore, the results showed that workers and architects were more likely to rate education and training as less important than personnel in managerial positions. Workers usually have less education and, therefore, may fail to recognize the importance of education and training for determining the safety climate. They may also perceive themselves as not requiring education and training due to the simple and repetitive nature of their work while failing to recognize the safety aspect of education and training. Architects are most concerned with the design aspects of construction. Therefore, they might perceive themselves as not requiring education and training regarding construction site safety because they generally engage in office-based work, with lower exposure to the construction activity on construction sites.

### 3.8. Communication

The results presented in Table 4 and Table S8 show that nationality, trade specialty, and occupation are the significant sociodemographic characteristics determining the importance of communication as a safety climate-influencing factor. Personnel of Egyptian, Syrian, Indian, and Filipino nationalities are more likely to rate communication as being more important than personnel of Pakistani nationality. Whereas the personnel group of Bangladeshi nationality was the only nationality group likely to rate communication as being less important than personnel of Pakistani nationality. Communication has been shown to influence the safety climate in many studies [21,46,47]. The nationality results in this study confirm this finding, as most nationalities gave higher ratings of this influencing factor. However, personnel of Bangladeshi nationality perceived the importance of communication as being lower than other nationalities. This result might be attributable to cultural differences; however, a deeper investigation is necessary. Moreover, the results showed that personnel in the trade specialties of bricklaying, plumbers, cement and concrete workers, blacksmith workers, and crane operators are more likely to rate communication as being less important than construction site personnel in the field of safety and quality control. Safety and quality personnel are expected to demonstrate higher ratings for communication than other specialty trades because safety and quality personnel are responsible for ensuring the effectiveness of construction site communication to ensure general safety. However, effective communication efforts should be directed toward the indicated worker groups to enhance the safety climate. Furthermore, the results showed that workers are more likely to rate communication as being less important than personnel in managerial positions, likely because managerial personnel who seek to implement strict safety measures at their construction sites tend to emphasize clear communication channels with their employees, based on a better understanding of communication barriers.

### 3.9. Workers’ Safety Commitment

The results presented in Table 4 and Table S9 reveal that trade specialty, education, and experience are the significant sociodemographic characteristics that determined the importance of workers’ safety commitment as a safety climate-influencing factor. The results show that carpenters and blacksmith workers were more likely to rate workers’ safety commitment as being less important than construction site personnel in the field of safety and quality control. Safety and quality control personnel are expected to rate this factor as being more important than both carpenters and blacksmith personnel due to their role in ensuring the safety of construction site employees and their belief in the importance of workers’ safety commitment. However, carpenters and blacksmith workers also tend to have lower education levels. Therefore, their view of the reduced importance of workers’ commitment to safety might be attributed to their limited background knowledge and awareness; however, this view remains alarming. Moreover, the results showed that personnel with educational levels ranging from illiterate to diploma holder were more likely to rate workers’ safety commitment as being less important than bachelor′s degree holders. As observed with previously discussed influencing factors, the level of education has a significant effect on the views of safety climate factors. Furthermore, personnel with 16–20 years of experience were more likely to rate workers’ safety commitment as being less important than the end-of-career personnel group, with 20 years or more of experience. Personnel with more experience appear to consider workers’ safety commitment to be more significant than those with less experience.

### 3.10. Workers’ Attitudes toward Health and Safety

The results presented in Table 4 and Table S10 show that age and experience were the significant sociodemographic characteristics that determined the importance of workers’ attitudes toward health and safety as a safety climate-influencing factor. The youngest personnel group, aged between 18 and 20 years, was the only group that was significantly likely to rate workers’ attitudes toward health and safety as being less important than the eldest personnel group, older than 50 years. This result is consistent with the results reported by Chen and Jin [48], which revealed that younger workers tend to have worse safety attitudes and overall perceptions than older workers. Moreover, the results show that the early-to-mid-career personnel groups, with 0 to 5 and 6 to 10 years of experience, are more likely to rate workers’ attitudes toward health and safety as being less important than the end-of-career personnel group, with 20 years or more of experience. Years of experience are associated with age and, thus, similar results are expected for this factor.

### 3.11. Workers’ Involvement

The results presented in Table 4 and Table S11 reveal that nationality, trade specialty, education, age, and experience were the significant sociodemographic characteristics that determined the importance of workers’ involvement as a safety climate-influencing factor. Personnel of Bangladeshi nationality were more likely to rate workers involvement’ as less important than personnel of Pakistani nationality. In contrast, personnel of Syrian, Egyptian, and Indian nationalities were more likely to rate this factor as more important than personnel of Pakistani nationality. These differences may be attributable to cultural background differences among these nationality groups. However, consistently with other findings, the personnel of a Bangladeshi nationality rated the importance of this influencing factor as opposite to the rating used by other nationalities; therefore, further analyses should be determined to understand the reasons for these wide differences in nationalities. Furthermore, the results showed that personnel classified as plumbers, bricklaying workers, and cement and concrete workers were more likely to rate workers’ involvement as being less important than construction site personnel classified as safety and quality control. These worker specialties are associated with lower education levels. Therefore, their lower rating of the importance of workers′ involvement may be due to their limited background knowledge, awareness, and lower safety leadership skills, which may affect their perception of the importance of worker involvement in safety. Moreover, the results showed that personnel who are illiterate or only have an elementary educational level were more likely to rate workers’ involvement as less important than those with bachelor′s degrees. As observed for other factors, this difference may be due to their limited background knowledge and lack of awareness. Furthermore, the results showed that the personnel group aged between 31 and 35 years was the only significant group likely to rate workers’ involvement as being less important than the eldest personnel group, aged 50 years or older. Additionally, early-to-mid-career personnel, with 0 to 5, 6 to 10, and 11 to 15 years of experience were more likely to rate workers’ involvement as being less important than the end-of-career personnel group, with 20 years or more of experience. These results demonstrated that this factor is more valued by older and more experienced construction personnel.

### 3.12. Supportive Environment

The results presented in Table 4 and Table S12 show that nationality and education were the significant sociodemographic characteristics that influenced the importance of a supportive environment for safety climate. Personnel of Bangladeshi nationality were more likely to rate a supportive environment as being less important than the personnel of Pakistani nationality. In contrast, the personnel group of Egyptian nationality was more likely to rate this factor as being more important than personnel of Pakistani nationality. This outcome matches the outcomes observed for other factors for which nationality was identified as a significant sociodemographic characteristic, and these differences may be attributed to cultural differences among those nationality groups. Moreover, the results showed that personnel who are illiterate or have elementary and intermediate educational levels were more likely to rate a supportive environment as being less important than bachelor′s degree holders. This outcome is also similar to the outcomes observed for other factors for which the education level was determined to be a significant sociodemographic characteristic, and this difference might also be attributed to their limited background knowledge and lack of awareness.

### 3.13. Competence

Finally, the results presented in Table 4 and Table S13 revealed that trade specialty and education were the significant sociodemographic characteristics that determined the importance of competence as a safety climate-influencing factor. The results showed that personnel classified as administration were more likely to rate competence as more important than construction site personnel classified as safety and quality control. The administration staff is office-based staff, with limited exposure to construction sites, whereas other groups belonging to specialty trades are on-site staff who may perceive themselves as sufficiently competent. As a result, unlike administration staff, on-site staff view competence as less influential for the safety climate. Moreover, the results showed that only personnel with elementary and intermediate educational levels were likely to rate competence as being less important than bachelor degree holders. This finding could be due to individuals with lower educational levels being more likely to work on simple repetitive tasks and therefore, perceiving competency as contributing less strongly to safety.

Generally, Table 4 shows that the educational level of construction site personnel was a significant sociodemographic characteristic that contributed to determining the importance of 11 safety climate-influencing factors, indicating the major role played by education in shaping the perceptions of construction site personnel concerning safety climate. Second to education, the nationality of construction site personnel was significant for seven safety climate-influencing factors, indicating the important roles played by different cultural backgrounds in shaping the perceptions of construction site personnel regarding safety climate. The third most consequential characteristic was trade specialty, which was significant for six safety climate-influencing factors, indicating the important roles that different trade specialties can play in changing the perceptions of construction site personnel regarding the safety climate. Similarly, the experience, age, and occupation had significant effects on perceptions of the safety climate. This overarching view demonstrates the need to design different safety climate enhancement strategies based on the specific sociodemographic composition of the construction site. Furthermore, these findings confirm the need for future research to examine the cultural, behavioral, and psychological differences among the multiple sociodemographic characteristics of construction site personnel and their effect on their perceptions of the safety climate.

## 4. Conclusions

The main objective of this study was to reveal the significant perceptions and multi-sociodemographic characteristics associated with construction site personnel regarding factors that influence the safety climate in the construction industry of Saudi Arabia. A total of 13 safety climate-influencing factors were regressed using the ordinal, logistic regression method, against six sociodemographic characteristics and associated subgroups. The results revealed significant sociodemographic characteristics and subgroups and their effects on the views of the importance of the contributions of different factors to the safety climate. The findings of this study revealed that, generally, education, nationality, trade specialty, experience, age, and occupation play major roles in shaping the perceptions of construction site personnel regarding the safety climate. These results may be associated with the different knowledge levels, cultural backgrounds, job nature, previous experiences, ages, and occupations of construction site personnel. This study provides a better understanding of the views held by construction site personnel concerning each of the safety climate-influencing factors and their revealed set of significant sociodemographic characteristics and the effect of their associated groups. Furthermore, this study can assist construction industry decision-makers, safety policy designers, government agencies, and stakeholders when designing better-targeted enhancement plans and strategies for improving the safety climate, based on the sociodemographic compositions of the personnel at construction sites.

The findings of this study demonstrated patterns among the collected dataset, which were representative of the construction industry in Saudi Arabia. Therefore, collecting more data from other spatial and temporal contexts represents an important future research direction. Moreover, ordinal, logistic regression was used in this study. Other prediction/classification techniques, such as machine learning methods, could be used in future research. Furthermore, studying the cultural, behavioral, and psychological differences among multiple sociodemographic characteristics associated with construction site personnel and determining their effect on their perceptions of the safety climate represents another future research direction.

## Figures and Tables

**Table 1:** Research sample sociodemographic characteristics (*N* = 401) ^1^.

Characteristic	Groups	*n*	%	Characteristic	Groups	*n*	%
Age (years)	18–20	2	0.50	Occupation	Worker	294	73.31
	21–25	51	12.72		Technician	15	3.74
	26–30	57	14.20		Supervisor	50	12.50
	31–35	89	22.20		Architect	3	0.75
	36–40	105	26.20		Engineer	29	7.20
	41–45	65	16.20		Manager	10	2.50
	Older than 50	9	2.24	Experience (years)	0–5	108	26.93
					6–10	134	33.41
Nationality	Sudanese	3	0.75		11–15	107	26.70
	Filipino	3	0.75		16–20	35	8.73
	Bangladeshi	6	1.50		More than 20	17	4.23
	Somali	7	1.75				
	Syrian	50	12.47	Trade specialty	Carpenter	100	24.93
	Indian	55	13.71		Blacksmith	82	20.45
	Yemeni	58	14.50		Bricklaying	39	9.72
	Egyptian	90	22.40		Painter	5	1.25
	Pakistani	129	32.17		Plumbing	34	8.50
					Cement and concrete	31	7.73
Education	Illiterate	70	17.45		Crane operator	7	1.75
	Elementary	68	16.96		Surveyor	9	2.24
	Intermediate	73	18.20		Mechanical	4	1.00
	Secondary	99	24.69		Architecture	3	0.75
	Diploma	31	7.70		Electrical	9	2.24
	Bachelor	60	15.00		Administration	15	3.74
					Civil	50	12.50
					Safety and quality control	13	3.20

^1^ Source: Mosly and Makki [37].

**Table 2:** Factors influencing the safety climate in the construction industry of Saudi Arabia.

Rank ^1,2^	Factors ^1,2^	Description ^1^
1	Supervision, guidance, and inspection	Assistance and assurance that a safety program is fully implemented on-site.
2	Appraisal of risks and hazards	The ability to assess risks and hazards.
3	Social security and health insurance	Providing workers with legal contracts and medical insurance.
4	Workmate influences	The influence of co-worker’s perceptions/practices on each other in terms of safety aspects.
5	Management safety justice	The degree of quality to which the management can deal fairly with workers involved in safety accidents.
6	Management commitment to safety	The priority level and care that management dedicates to workers’ safety.
7	Education and training	Information, instructions, and learning materials provided to workers on safety.
8	Communication	Effective communication by management and workers’ feedback.
9	Workers’ safety commitment	The priority level and care that workers dedicate to their own and others’ safety on site.
10	Workers’ attitude toward health and safety	The perception of the worker towards aspects of health and safety, and the willingness degree to risk taking.
11	Workers’ involvement	Involvement and contribution of workers in safety activities.
12	Supportive environment	Overall safety trust and support between a group of employees.
13	Competence	The general background of workers’ knowledge, training, qualification, and skills.

^1^ Source: Mosly and Makki [37]. ^2^ Factors are ranked based on the significance of their correlations with the overall safety climate, as perceived by construction site personnel.

**Table 3:** Sociodemographic characteristics and reference groups assignment criteria.

Sociodemographic Characteristic	Type	Reference Group	Assignment Criterion(a)
Age	Ordinal	Older than 50	Highest order ^1^
Nationality	Nominal	Pakistani	Largest nationality sample size
Education	Ordinal	Bachelor	Highest order ^1^
Occupation	Nominal	Manager	Highest authority/education
Experience	Ordinal	More than 20	Highest order ^1^
Trade specialty	Nominal	Safety and quality control	Daily work is directly related to safety/highest education

^1^ Last category on an ascending scale.

**Table 4:** Results summary of statistically significant sociodemographic characteristics and associated groups versus safety climate-influencing factors.

Factor ^1^	Age ^2^	Nationality ^2^	Education ^2^	Occupation ^2^	Experience ^2^	Trade Specialty ^2^
Supervision, guidance, and inspection		Egyptian ^H^	Illiterate ^L^			Carpenter ^L^
		Bangladeshi ^L^	Elementary ^L^			Plumbing ^L^
			Intermediate ^L^			
			Secondary ^L^			
			Diploma ^L^			
Appraisal of risks and hazards	18–20 ^L^		Illiterate ^L^			
			Elementary ^L^			
			Intermediate ^L^			
			Secondary ^L^			
Social security and health insurance			Illiterate ^L^			
Workmate influences		Bangladeshi ^L^	Illiterate ^L^			
		Yemeni ^L^	Elementary ^L^			
		Egyptian ^H^	Intermediate ^L^			
		Filipino ^H^	Secondary ^L^			
Management safety justice			Illiterate ^L^		11–15 ^H^	Administration ^H^
			Elementary ^L^			Plumbing ^H^
			Intermediate ^L^			
			Secondary ^L^			
			Diploma ^L^			
Management commitment to safety		Bangladeshi ^L^	Illiterate ^L^			
		Egyptian ^H^	Elementary ^L^			
			Intermediate ^L^			
			Secondary ^L^			
			Diploma ^L^			
Education and training		Bangladeshi ^L^	Illiterate ^L^	Worker ^L^		
		Somali^L^	Elementary ^L^	Architect ^L^		
		Egyptian ^H^	Intermediate			
			Secondary ^L^			
			Diploma ^L^			
Communication		Egyptian ^H^		Worker ^L^		Bricklaying ^L^
		Syrian ^H^				Plumbing ^L^
		Indian ^H^				Cement and concrete ^L^
		Filipino ^H^				Blacksmith ^L^
		Bangladeshi ^L^				Crane operator ^L^
Workers’ safety commitment			Illiterate ^L^		16–20 ^L^	Carpenter ^L^
			Elementary ^L^			Blacksmith ^L^
			Intermediate ^L^			
			Secondary ^L^			
			Diploma ^L^			
Workers’ attitude toward health and safety	18–20 ^L^				0–5 ^L^	
					6–10 ^L^	
Workers’ involvement	31–35 ^L^	Bangladeshi ^L^	Illiterate ^L^		0–5 ^L^	Plumbing ^L^
		Syrian ^H^	Elementary ^L^		6–10 ^L^	Bricklaying ^L^
		Egyptian ^H^			11–15 ^L^	Cement and concrete ^L^
		Indian ^H^				
Supportive environment		Bangladeshi ^L^	Illiterate ^L^			
		Egyptian ^H^	Elementary ^L^			
			Intermediate ^L^			
Competence			Elementary ^L^			Administration ^H^
			Intermediate ^L^			

^1^ Factors are ranked as presented in Table 2. ^2^ The assigned reference group for this sociodemographic characteristic can be found in Table 3. ^H^ Odds ratio and statistical significance indicate that this sociodemographic category is most likely to rate the importance of the safety climate factor higher than the reference group of the pertaining sociodemographic characteristic. ^L^ Odds ratio and statistical significance indicate that this sociodemographic category is most likely to rate the importance of the safety climate factor lower than the reference group of the pertaining sociodemographic characteristic.

## Data Availability

The data presented in this study are available on request from the corresponding author. The data are not publicly available due to privacy and ethical restrictions.

## Supplementary Materials

The following are available online at https://www.mdpi.com/1660-4601/18/4/1674/s1, Table S1: Statistically significant sociodemographic characteristics and their associated subgroups to the safety climate influencing factor: Supervision, guidance, and inspection, Table S2: Statistically significant sociodemographic characteristics and their associated subgroups to the safety climate influencing factor: Appraisal of risks and hazards, Table S3: Statistically significant sociodemographic characteristics and their associated subgroups to the safety climate influencing factor: Social security and health insurance, Table S4: Statistically significant sociodemographic characteristics and their associated subgroups to the safety climate influencing factor: Workmate influences, Table S5: Statistically significant sociodemographic characteristics and their associated subgroups to the safety climate influencing factor: Management safety justice, Table S6: Statistically significant sociodemographic characteristics and their associated subgroups to the safety climate influencing factor: Management commitment to safety, Table S7: Statistically significant sociodemographic characteristics and their associated subgroups to the safety climate influencing factor: Education and training, Table S8: Statistically significant sociodemographic characteristics and their associated subgroups to the safety climate influencing factor: Communication, Table S9: Statistically significant sociodemographic characteristics and their associated subgroups to the safety climate influencing factor: Workers safety commitment, Table S10: Statistically significant sociodemographic characteristics and their associated subgroups to the safety climate influencing factor: Workers attitude toward health and safety, Table S11: Statistically significant sociodemographic characteristics and their associated subgroups to the safety climate influencing factor: Workers involvement, Table S12: Statistically significant sociodemographic characteristics and their associated subgroups to the safety climate influencing factor: Supportive environment, Table S13: Statistically significant sociodemographic characteristics and their associated subgroups to the safety climate influencing factor: Competence.

## References

[1] Lingard H., Cooke T., Blismas N. (2009). Group-level safety climate in the Australian construction industry: Within-group homogeneity and between-group differences in road construction and maintenance. Constr. Manag. Econ..

[2] Andersen L., Nørdam L., Joensson T., Kines P., Nielsen K. (2018). Social identity, safety climate and self-reported accidents among construction workers. Constr. Manag. Econ..

[3] Hassan C., Basha O., Hanafi W. (2007). Perception of Building Construction Workers Towards Safety, Health and Environment. J. Eng. Sci. Technol..

[4] Ricci F., Modenese A., Bravo G., Pasquale F., Ferrari D., Bello M., Carozza L., Longhi F., Favero G., Soddu S. (2020). Ethnic Background and Risk Perception in Construction Workers: Development and Validation of an Exploratory Tool. Int. J. Occup. Med. Environ. Health.

[5] Gao R., Chan A., Utama W., Zahoor H. (2017). Workers’ Perceptions of Safety Climate in International Construction Projects: Effects of Nationality, Religious Belief, and Employment Mode. J. Constr. Eng. Manag..

[6] Han Y., Feng Z., Zhang J., Jin R., Aboagye-Nimo E. (2019). Employees’ Safety Perceptions of Site Hazard and Accident Scenes. J. Constr. Eng. Manag..

[7] Han Y., Jin R., Wood H., Yang T. (2019). Investigation of Demographic Factors in Construction Employees’ Safety Perceptions. KSCE J. Civ. Eng..

[8] Shen Y., Koh T., Rowlinson S., Bridge A. (2015). Empirical Investigation of Factors Contributing to the Psychological Safety Climate on Construction Sites. J. Constr. Eng. Manag..

[9] Elmoujaddidi F., Bachir A. (2020). Perceived risk, safety climate and safety behavior on Moroccan construction sites. Int. J. Occup. Saf. Ergon..

[10] Gillen M., Baltz D., Gassel M., Kirsch L., Vaccaro D. (2002). Perceived safety climate, job demands, and coworker support among union and nonunion injured construction workers. J. Saf. Res..

[11] Man K., Chan A. Influence of Personality and Safety Climate on Risk Perception of Hong Kong Construction Workers. Proceedings of the International MultiConference of Engineers and Computer Scientists 2018.

[12] Mosly I. (2019). Factors Influencing Safety Climate in the Construction Industry: A Review. Int. J. Constr. Eng. Manag..

[13] Pandit B., Albert A., Patil Y., Al-Bayati A. (2019). Impact of safety climate on hazard recognition and safety risk perception. Saf. Sci..

[14] Choudhry R., Fang D., Lingard H. (2009). Measuring Safety Climate of a Construction Company. J. Constr. Eng. Manag..

[15] Stoilkovska B., Pančovska V., Mijoski G. (2015). Relationship of safety climate perceptions and job satisfaction among employees in the construction industry: The moderating role of age. Int. J. Occup. Saf. Ergon..

[16] Gyekye S. (2005). Workers’ Perceptions of Workplace Safety and Job Satisfaction. Int. J. Occup. Saf. Ergon..

[17] Shen Y., Ju C., Koh T., Rowlinson S., Bridge A. (2017). The Impact of Transformational Leadership on Safety Climate and Individual Safety Behavior on Construction Sites. Int. J. Environ. Res. Public Health.

[18] Glendon A., Litherland D. (2001). Safety climate factors, froup differences and safety behaviour in road construction. Saf. Sci..

[19] Brondino M., Silva S., Pasini M. (2012). Multilevel approach to organizational and group safety climate and safety performance: Co-workers as the missing link. Saf. Sci..

[20] Xia N., Xie Q., Hu X., Wang X., Meng H. (2020). A dual perspective on risk perception and its effect on safety behavior: A moderated mediation model of safety motivation, and supervisor’s and coworkers’ safety climate. Accid. Anal. Prev..

[21] Pandit B., Albert A., Patil Y., Al-Bayati A. (2019). Fostering Safety Communication among Construction Workers: Role of Safety Climate and Crew-Level Cohesion. Int. J. Environ. Res. Public Health.

[22] Lingard H., Cooke T., Blismas N. (2012). Do Perceptions of Supervisors’ Safety Responses Mediate the Relationship between Perceptions of the Organizational Safety Climate and Incident Rates in the Construction Supply Chain?. J. Constr. Eng. Manag..

[23] Lyu S., Hon C., Chan A., Wong F., Javed A. (2018). Relationships among Safety Climate, Safety Behavior, and Safety Outcomes for Ethnic Minority Construction Workers. Int. J. Environ. Res. Public Health.

[24] Chan A., Javed A., Wong F., Hon C., Lyu S. (2017). Evaluating the Safety Climate of Ethnic Minority Construction Workers in Hong Kong. J. Prof. Issues Eng. Educ. Pract..

[25] Arcury T., Mills T., Marı’n A., Summers P., Quandt S., Rushing J., Lang W., Grzywacz J. (2012). Work Safety Climate and Safety Practices Among Immigrant Latino Residential Construction Workers. Am. J. Ind. Med..

[26] Chan A., Wong F., Hon C., Lyu S., Javed A. (2017). Investigating ethnic minorities’ perceptions of safety climate in the construction industry. J. Saf. Res..

[27] Cigularov K., Lancaster P., Chen P., Gittleman J., Haile E. (2013). Measurement equivalence of a safety climate measure among Hispanic and White Non-Hispanic construction workers. Saf. Sci..

[28] Welton M., DeJoy D., Castellanos M., Ebell M., Shen Y., Robb S. (2018). Ethnic Disparities of Perceived Safety Climate Among Construction Workers in Georgia, 2015. J. Racial Ethn. Health Disparities.

[29] Luria G., Yagil D. (2010). Safety perception referents of permanent and temporary employees: Safety climate boundaries in the industrial workplace. Accid. Anal. Prev..

[30] Zhang R., Lingard H., Nevin S. (2015). Development and validation of a multilevel safety climate measurement tool in the construction industry. Constr. Manag. Econ..

[31] Oah S., Na R., Moon K. (2018). The Influence of Safety Climate, Safety Leadership, Workload, and Accident Experiences on Risk Perception: A Study of Korean Manufacturing Workers. Saf. Health Work.

[32] Cigularov K., Adams S., Gittleman J., Haile E., Chen P. (2013). Measurement equivalence and mean comparisons of a safety climate measure across construction trades. Accid. Anal. Prev..

[33] Chen W., Merrett H., Huang Y., Lu S., Sun W., Li Y. (2019). Exploring the Multilevel Perception of Safety Climate on Taiwanese Construction Sites. Sustainability.

[34] Marína L., Lipscomb H., Cifuentes M., Punnett L. (2019). Perceptions of safety climate across construction personnel: Associations with injury rates. Saf. Sci..

[35] Pinion C., Klyza J., Brewer S., Douphrate D. (2018). North American Engineering, Procurement, Fabrication and ConstructionWorker Safety Climate Perception Affected by Job Position. Safety.

[36] Mosly I. (2015). Safety Performance in the Construction Industry of Saudi Arabia. Int. J. Constr. Eng. Manag..

[37] Mosly I., Makki A. (2020). Safety Climate Perceptions in the Construction Industry of Saudi Arabia: The Current Situation. Int. J. Environ. Res. Public Health.

[38] Makki A., Mosly I. (2020). Determinants for Safety Climate Evaluation of Construction Industry Sites in Saudi Arabia. Int. J. Environ. Res. Public Health.

[39] Makki A., Mosly I. (2021). Predicting the Safety Climate in Construction Sites of Saudi Arabia: A Bootstrapped Multiple Ordinal Logistic Regression Modeling Approach. Appl. Sci..

[40] O’Connell A. (2006). Logistic Regression Models for Ordinal Response Variables.

[41] Harrell F. (2015). Ordinal Logistic Regression. Regression Modeling Strategies.

[42] Field A. (2018). Discovering Statistics Using IBM SPSS Statistics.

[43] IBM (2015). IBM SPSS Statistics for Windows.

[44] Lingard H., Cooke T., Blismas N. (2011). Coworkers’ response to occupational health and safety An overlooked dimension of group-level safety climate in the construction industry?. Eng. Constr. Archit. Manag..

[45] Chen Q., Jin R. (2013). Multilevel Safety Culture and Climate Survey for Assessing New Safety Program. J. Constr. Eng. Manag..

[46] Greeff W. (2017). The role of communication in managing the safety climate of construction site environments. Communicatio.

[47] Zamani V., Banihashemi S., Abbasi A. (2020). How can communication networks among excavator crew members in construction projects affect the relationship between safety climate and safety outcomes?. Saf. Sci..

[48] Chen Q., Jin R. (2015). A comparison of subgroup construction workers’ perceptions of a safety program. Saf. Sci..

