# Supplementary material for: The Effects of Multi-Sociodemographic Characteristics of Construction Sites Personnel on Perceptions of Safety Climate-Influencing Factors: The Construction Industry in Saudi Arabia
Source: Int J Environ Res Public Health. 2021 Feb 9;18(4):1674. doi: 10.3390/ijerph18041674 (PMC7916194; doi:10.3390/ijerph18041674)
Supplement: Supplementary file 1 [file ijerph-18-01674-s001.pdf]

The Effects of Multi-Sociodemographic Characteristics of Construction Sites Personnel on Perceptions of Safety Climate-Influencing Factors: The Construction Industry in Saudi Arabia

Ibrahim Mosly [1,*] and Anas A. Makki [2]

Table S1. Statistically significant sociodemographic characteristics and their associated subgroups to the safety climate influencing factor: Supervision, guidance, and inspection**.**

Factor	Sociodemographic Charachteristic			Omnibus Test	Pseudo R^2	Test of Parallel Lines			Parameter Estimates			
**DV**	**IV**	**Reference Category**	***df***	**Likelihood Ratio χ^2**	**Nagelkerke's R^2**	**-2 Log Likelihood**	***df***	**χ^2**	**Location**	**β**	**Exp(β) [a]**	**Wald χ^2 [b]**
Supervision, guidance, and inspection	Trade Specialty	Safety & Quality Control	13	35.788***	.097	105.196	26	3.460'	Carpenter	-1.726	0.178	6.427*
									Plumbing	-1.446	0.236	3.861*
	Education	Bachelor	5	31.474***	.086	56.986	10	10.670'	Illiterate	-1.416	0.243	14.084***
									Elementary	-1.698	0.183	20.086***
									Intermediate	-1.702	0.182	20.457***
									Secondary	-0.970	0.379	7.286**
									Diploma	-0.925	0.397	3.922*
	Nationality	Pakistani	8	22.156**	.061	56.860	16	23.037'	Egyptian	0.865	2.376	10.086***
									Bangladesh	-1.920	0.147	4.544*

' $p > .05$, *$p < .05$, **$p < .01$, ***$p < .001$. [a] 95% Wald confidence interval for Exp(β). [b] df=1.

Table S2. Statistically significant sociodemographic characteristics and their associated subgroups to the safety climate influencing factor: Appraisal of risks and hazards.

Factor	Sociodemographic Charachteristic			Omnibus Test	Pseudo R^2	Test of Parallel Lines			Parameter Estimates			
**DV**	**IV**	**Reference Category**	***df***	**Likelihood Ratio χ^2**	**Nagelkerke's R^2**	**-2 Log Likelihood**	***df***	**χ^2**	**Location**	**β**	**Exp(β) [a]**	**Wald χ^2 [b]**
Appraisal of risks and hazards	Education	Bachelor	5	27.551***	.073	65.613	10	15.206'	Illiterate	-1.599	0.202	21.302***
									Elementary	-1.404	0.246	16.133***
									Intermediate	-1.263	0.283	13.810***
									Secondary	-1.065	0.345	11.114***
	Age	More than 50	7	18.264*	.049	68.605	14	12.114'	18-20	-3.616	0.027	6.154*

' $p > .05$, *$p < .05$, ***$p < .001$. [a] 95% Wald confidence interval for Exp(β). [b] df=1.

Table S3. Statistically significant sociodemographic characteristics and their associated subgroups to the safety climate influencing factor: Social security and health insurance.

Factor	Sociodemographic Charachteristic		Omnibus Test		Pseudo R^2	Test of Parallel Lines			Parameter Estimates			
DV	IV	Reference Category	*df*	Likelihood Ratio χ^2	Nagelkerke's R^2	-2 Log Likelihood	*df*	χ^2	Location	β	Exp(β) [a]	Wald χ^2 [b]
Social security and health insurance	Education	Bachelor	5	14.339*	.039	72.021	15	9.994'	Illiterate	-1.178	0.308	11.965***

' p >.05, *p < .05, ***p < .001. [a] 95% Wald confidence interval for Exp(β). [b] df=1.

Table S4. Statistically significant sociodemographic characteristics and their associated subgroups to the safety climate influencing factor: Workmate influences.

Factor	Sociodemographic Charachteristic		Omnibus Test		Pseudo R^2	Test of Parallel Lines			Parameter Estimates			
DV	IV	Reference Category	*df*	Likelihood Ratio χ^2	Nagelkerke's R^2	-2 Log Likelihood	*df*	χ^2	Location	β	Exp(β) [a]	Wald χ^2 [b]
Workmate influences	Nationality	Pakistani	8	54.722***	.136	79.039	24	20.766'	Bangladesh	-3.149	0.043	11.081***
									Yemen	-0.951	0.386	10.574***
									Egyptian	0.661	1.937	6.930**
									Philippine	2.788	16.255	5.354**
	Education	Bachelor	5	38.178***	.097	76.345	15	16.791'	Illiterate	-1.86	0.156	30.64***
									Elementary	-1.182	0.307	12.666***
									Intermediate	-0.871	0.418	7.447**
									Secondary	-0.844	0.430	7.740**

' p >.05 **p < .01, ***p < .001. [a] 95% Wald confidence interval for Exp(β). [b] df=1.

Table S5. Statistically significant sociodemographic characteristics and their associated subgroups to the safety climate influencing factor: Management safety justice.

Factor	Sociodemographic Charachteristic		Omnibus Test		Pseudo R^2	Test of Parallel Lines			Parameter Estimates			
DV	IV	Reference Category	*df*	Likelihood Ratio χ^2	Nagelkerke's R^2	-2 Log Likelihood	*df*	χ^2	Location	β	Exp(β) [a]	Wald χ^2 [b]
Management safety justice	Trade Specialty	Safety & Quality Control	13	61.61***	.154	103.051	39	35.334'	Administration	2.221	9.215	7.215**
									Plumbing	1.382	3.983	4.822*
	Education	Bachelor	5	25.822***	.067	82.481	15	17.607'	Illiterate	-1.109	0.330	10.384***
									Elementary	-1.513	0.220	19.000***
									Intermediate	-1.405	0.245	17.707***
									Secondary	-0.823	0.439	6.682**
									Diploma	-1.082	0.339	6.706**
	Experience	More than 20	4	17.910***	.047	63.189	12	15.079'	11-15	0.920	2.509	4.016*

' p >.05, *p < .05, **p < .01, ***p < .001. [a] 95% Wald confidence interval for Exp(β). [b] df=1.

Table S6. Statistically significant sociodemographic characteristics and their associated subgroups to the safety climate influencing factor: Management commitment to safety.

Factor	Sociodemographic Charachteristic		Omnibus Test		Pseudo R^2	Test of Parallel Lines			Parameter Estimates			
DV	IV	Reference Category	*df*	Likelihood Ratio χ^2	Nagelkerke's R^2	-2 Log Likelihood	*df*	χ^2	Location	β	Exp(β) [a]	Wald χ^2 [b]
Management commitment to safety	Education	Bachelor	5	19.565***	.053	65.277	15	19.492ˈ	Illiterate	-1.303	0.272	12.811***
									Elementary	-1.298	0.273	12.93***
									Intermediate	-1.218	0.296	11.475***
									Secondary	-0.891	0.410	6.805**
									Diploma	-1.326	0.266	8.852**
	Nationality	Pakistani	8	15.733*	.046	64.327	24	23.737ˈ	Bangladesh	-1.851	0.157	5.569*
									Egyptian	0.517	1.677	3.957*

ˈ p >.05, *p < .05, **p < .01, ***p < .001. [a] 95% Wald confidence interval for Exp(β). [b] *df*=1.

Table S7. Statistically significant sociodemographic characteristics and their associated subgroups to the safety climate influencing factor: Education and training.

Factor	Sociodemographic Charachteristic		Omnibus Test		Pseudo R^2	Test of Parallel Lines			Parameter Estimates			
DV	IV	Reference Category	*df*	Likelihood Ratio χ^2	Nagelkerke's R^2	-2 Log Likelihood	*df*	χ^2	Location	β	Exp(β) [a]	Wald χ^2 [b]
Education and training	Nationality	Pakistani	8	50.446***	.133	62.883	24	22.737ˈ	Bangladesh	-3.639	0.026	18.555***
									Somalia	-2.417	0.089	10.190***
									Egyptian	0.592	1.808	4.982*
	Education	Bachelor	5	24.562***	.067	63.376	15	13.242ˈ	Illiterate	-1.283	0.277	12.293***
									Elementary	-0.719	0.487	3.872*
									Intermediate	-1.505	0.222	17.695***
									Secondary	-0.740	0.477	4.640*
									Diploma	-1.328	0.265	9.409**
	Occupation	Manager	5	11.899*	.033	44.197	15	7.959ˈ	Worker	-2.31	0.099	4.767*
									Architect	-2.943	0.053	3.935*

ˈ p >.05, *p < .05, **p < .01, ***p < .001. [a] 95% Wald confidence interval for Exp(β). [b] *df*=1.

Table S8. Statistically significant sociodemographic characteristics and their associated subgroups to the safety climate influencing factor: Communication.

Factor	Sociodemographic Charachteristic		Omnibus Test		Pseudo R^2	Test of Parallel Lines			Parameter Estimates			
DV	IV	Reference Category	*df*	Likelihood Ratio χ^2	Nagelkerke's R^2	-2 Log Likelihood	*df*	χ^2	Location	β	Exp(β) [a]	Wald χ^2 [b]
Communication	Nationality	Pakistani	8	69.287***	.170	75.799	24	19.606ˈ	Egyptian	1.316	3.729	25.422***
									Syrian	1.411	4.098	20.25***
									Indian	1.160	3.190	14.385***

									Philippine	3.490	32.773	8.242**
									Bangladesh	-1.616	0.199	4.116*
	Trade Specialty	Safety & Quality Control	13	47.303***	.119	107.913	39	43.629ʹ	Bricklaying	-2.310	0.099	12.944***
									Plumbing	-1.914	0.148	8.759**
									Cement & concrete	-1.592	0.203	5.929*
									Blacksmith	-1.365	0.255	5.143*
									Crane Operator	-1.785	0.168	4.651*
	Occupation	Manager	5	26.241***	.068	54.758	15	16.290ʹ	Worker	-1.824	0.161	9.012**

ʹ *p* >.05, **p* < .05, ***p* < .01, ****p* < .001. [a] 95% Wald confidence interval for Exp(β). [b] *df*=1.

Table S9. Statistically significant sociodemographic characteristics and their associated subgroups to the safety climate influencing factor: Workers safety commitment.

Factor	Sociodemographic Charachteristic		Omnibus Test		Pseudo R^2	Test of Parallel Lines			Parameter Estimates			
**DV**	**IV**	**Reference Category**	***df***	**Likelihood Ratio χ^2**	**Nagelkerke's R^2**	**-2 Log Likelihood**	***df***	**χ^2**	**Location**	**β**	**Exp(β) [a]**	**Wald χ^2 [b]**
Workers safety commitment	Trade Specialty	Safety & Quality Control	13	65.071***	.163	79.12	39	46.893ʹ	Carpenter	-1.933	0.145	10.521***
									Blacksmith	-1.598	0.202	7.059**
	Education	Bachelor	5	30.813***	.080	72.194	15	19.802ʹ	Illiterate	-1.591	0.204	20.736***
									Elementary	-1.688	0.185	23.012***
									Intermediate	-1.326	0.266	15.380***
									Secondary	-1.279	0.278	15.352***
									Diploma	-0.942	0.390	4.699*
	Experience	More than 20	4	11.268*	.030	66.161	12	13.099ʹ	16-20	-1.05	0.35	3.995*

ʹ *p* >.05, **p* < .05, ***p* < .01, ****p* < .001. [a] 95% Wald confidence interval for Exp(β). [b] *df*=1.

Table S10. Statistically significant sociodemographic characteristics and their associated subgroups to the safety climate influencing factor: Workers attitude toward health and safety.

Factor	Sociodemographic Charachteristic		Omnibus Test		Pseudo R^2	Test of Parallel Lines			Parameter Estimates			
**DV**	**IV**	**Reference Category**	***df***	**Likelihood Ratio χ^2**	**Nagelkerke's R^2**	**-2 Log Likelihood**	***df***	**χ^2**	**Location**	**β**	**Exp(β) [a]**	**Wald χ^2 [b]**
Workers attitude toward health and safety	Age	More than 50	7	27.650***	.074	74.779	21	17.692ʹ	18-20	-2.973	0.051	4.274*
	Experience	More than 20	4	17.545**	.047	57.434	12	18.886ʹ	0-5	-1.467	0.231	9.066**
									6-10	-1.363	0.256	8.004**

ʹ *p* >.05, **p* < .05, ***p* < .01, ****p* < .001. [a] 95% Wald confidence interval for Exp(β). [b] *df*=1.

Table S11. Statistically significant sociodemographic characteristics and their associated subgroups to the safety climate influencing factor: Workers involvement.

Factor	Sociodemographic Charachteristic		Omnibus Test		Pseudo R^2	Test of Parallel Lines			Parameter Estimates			
**DV**	**IV**	**Reference Category**	***df***	**Likelihood Ratio χ^2**	**Nagelkerke's R^2**	**-2 Log Likelihood**	***df***	**χ^2**	**Location**	**β**	**Exp(β) [a]**	**Wald χ^2 [b]**
	Nationality	Pakistani	8	52.835***	.133	73.419	24	25.876ʹ	Bangladesh	-3.183	0.041	15.029***

									Syrian	1.325	3.761	18.275***
									Egyptian	0.775	2.172	9.148**
									Indian	0.628	1.874	4.584*
Workers involvement	Trade Specialty	Safety & Quality Control	13	52.457***	.132	101.577	39	40.55'	Plumbing	-1.955	0.142	9.746**
									Bricklaying	-1.415	0.243	5.241*
									Cement & concrete	-1.242	0.289	3.874*
	Education	Bachelor	5	22.351***	.058	95.040	15	1.576'	Illiterate	-1.278	0.279	14.856***
									Elementary	-0.827	0.437	6.465*
	Age	More than 50	7	18.339*	.048	82.616	21	16.654'	31-35	-1.744	0.175	5.496*
	Experience	More than 20	4	14.269**	.038	63.703	12	6.950'	0-5	-1.599	0.202	10.078**
									6-10	-1.215	0.297	6.014*
									11-15	-1.177	0.308	5.502*

' p >.05, *p < .05, **p < .01, ***p < .001. [a] 95% Wald confidence interval for Exp(β). [b] df=1.

Table S12. Statistically significant sociodemographic characteristics and their associated subgroups to the safety climate influencing factor: Supportive environment.

Factor	Sociodemographic Charachteristic		Omnibus Test		Pseudo R^2	Test of Parallel Lines			Parameter Estimates			
DV	IV	Reference Category	df	Likelihood Ratio χ^2	Nagelkerke's R^2	-2 Log Likelihood	df	χ^2	Location	β	Exp(β) [a]	Wald χ^2 [b]
Supportive environment	Nationality	Pakistani	8	27.761***	.073	73.154	24	21.329'	Bangladesh	-2.530	0.080	11.422***
									Egyptian	0.693	1.999	7.162**
	Education	Bachelor	5	14.589*	.039	72.519	15	18.908'	Illiterate	-0.883	0.414	7.153**
									Elementary	-0.991	0.371	8.631**
									Intermediate	-0.967	0.38	8.827**

' p >.05, *p < .05, **p < .01, ***p < .001. [a] 95% Wald confidence interval for Exp(β). [b] df=1.

Table S13. Statistically significant sociodemographic characteristics and their associated subgroups to the safety climate influencing factor: Competence.

Factor	Sociodemographic Charachteristic		Omnibus Test		Pseudo R^2	Test of Parallel Lines			Parameter Estimates			
DV	IV	Reference Category	df	Likelihood Ratio χ^2	Nagelkerke's R^2	-2 Log Likelihood	df	χ^2	Location	β	Exp(β) [a]	Wald χ^2 [b]
Competence	Trade Specialty	Safety & Quality Control	13	24.562*	.065	99.200	39	32.385'	Administration	1.772	5.882	5.809*
	Education	Bachelor	5	15.596**	.042	71.707	15	12.776'	Elementary	-1.039	0.354	9.630**
									Intermediate	-0.982	0.375	8.962**

' p >.05, *p < .05, **p < .01. [a] 95% Wald confidence interval for Exp(β). [b] df=1.
